# Reconstructing recent population history while mapping rare variants using haplotypes

**DOI:** 10.1038/s41598-019-42385-6

**Published:** 2019-04-10

**Authors:** Ural Yunusbaev, Albert Valeev, Milyausha Yunusbaeva, Hyung Wook Kwon, Reedik Mägi, Mait Metspalu, Bayazit Yunusbayev

**Affiliations:** 1 Ufa Federal Research Center of the Russian Academy of Sciences, Institute of Biochemistry and Genetics, Ufa, 450054 Russia; 2 Bashkir State Pedagogical University n. a. M. Akmulla, Department of Genetics, Ufa, 450000 Russia; 3 University of Tartu, Institute of Genomics, Tartu, 51010 Estonia; 4 Incheon National University, College of Life Science and Bioengineering, Incheon, 22012 South Korea

## Abstract

Haplotype-based methods are a cost-effective alternative to characterize unobserved rare variants and map disease-associated alleles. Moreover, they can be used to reconstruct recent population history, which shaped distribution of rare variants and thus can be used to guide gene mapping studies. In this study, we analysed Illumina 650 k genotyped dataset on three underrepresented populations from Eastern Europe, where ancestors of Russians came into contact with two indigenous ethnic groups, Bashkirs and Tatars. Using the IBD mapping approach, we identified two rare IBD haplotypes strongly enriched in asthma patients of distinct ethnic background. We reconstructed recent population history using haplotype-based methods to reconcile this contradictory finding. Our ChromoPainter analysis showed that these haplotypes each descend from a single ancestor coming from one of the ethnic groups studied. Next, we used DoRIS approach and showed that source populations for patients exchanged recent (<60 generations) asymmetric gene flow, which supported the ChromoPainter-based scenario that patients share haplotypes through inter-ethnic admixture. Finally, we show that these IBD haplotypes overlap with asthma-associated genomic regions ascertained in European population. This finding is consistent with the fact that the two donor populations for the rare IBD haplotypes: Russians and Tatars have European ancestry.

## Introduction

Low frequency (1% < MAF < 5%) and rare genetic variants (<1%) evolved recently and tend to have more deleterious effect^1^. While such variants may play an important role in the heritability of complex traits^2,3^, their effect remains largely uncharacterized. Accurate detection of rare variants requires extremely large samples (>10000) and costly high-coverage resequencing^4^. Therefore, there is a need for cost-effective methods to study rare variants in populations that are underrepresented in large-scale full genome sequencing projects. Chip-genotyped SNP datasets and rare haplotypes (<1%) constructed from them can be used as proxies for rare variants^5,6^. For populations that are not present in large-scale re-sequencing projects, use of haplotypes as proxies for rare variants is the only available option currently.

Distribution of rare variants has been shaped by more recent demographic events (5000–10000 years ago) in human population history^7^. Therefore, when mapping rare variants, knowledge about the recent demographic history for each studies population is essential^7^. In this regard, haplotype-based methods offer a rich arsenal of methods designed to reconstruct recent population history^8^.

**Figure 1:**
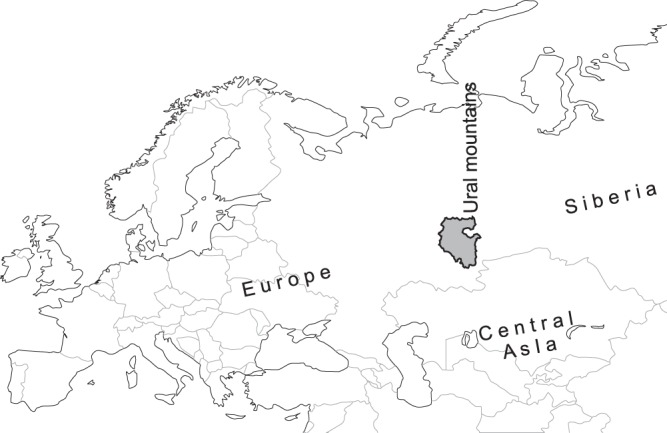
Population source region in the context of the Eurasian continent. The territory of the Republic of Bashkortostan is shown in grey.

In this study, we focus on underrepresented populations (Table 1) from Eastern Europe, the region that borders Central Asia and Siberia (Fig. 1). This region denoted as the Volga-Ural region has been a historical crossroad for human migrations and admixture^9,10^. It represents a useful model to understand the effect of recent complex population history on the distribution of rare haplotypes that serve here as a proxy for rare variants. Genome-wide data for our samples were retrieved from a previously published dataset^11^, and here we briefly describe background information relevant for our study. Patients and healthy controls were recruited from the Republic of Bashkortostan (Fig. 1), which geographically represent easternmost European Russia. Three ethnic groups currently represent the majority of the population in this region: Bashkirs, Russians, and Tatars, each amounting roughly 1/3 of the total population (total census size ~4.5 million people). Of these, Bashkirs and Tatars are indigenous to the region and speak Turkic languages. Although these populations have cultural affinities to Central Asian Turkic-speaking peoples, their genetic makeup is predominantly of European ancestry with varying proportion of genetic contributions from Central Asian and Siberian populations^9,10^. Russians are Slavic-speaking people with genetic affinities to Central European populations^12^ that expanded eastward from their historical lands only a few hundred years ago^13^.

**Table 1:** Study populations.

Ethnic group	Dataset				Сensus*	
	Patients	Controls	Total, n	Total, %	Total, n	Total, %
Russians	141	144	285	42	1432906	35
Tatars	120	109	229	34	1009295	25
Bashkirs	69	90	159	24	1172287	29
Others	—	—	—	—	457804	11
Total	330	343	673	100	4072292	100

*Official website of the Russian Census 2010 data^66^.

In this study, we examine the distribution of identical-by-descent (IBD) haplotypes among asthma patients and healthy controls in order to characterise unobserved rare variants enriched in patients. This approach has sufficient power to map rare variants using moderately sized samples of affected individuals^6,7^. It is expected that long (>1 cM) and rare (<1%) IBD tracts found among affected individuals most likely carry a disease risk variant because chances of finding even a small number of shared IBD haplotype among multiple unrelated individuals is very small^6^. We use the term IBD haplotype throughout the text when haplotypes have high support for being IBD as inferred using the refined IBD algorithm^14^, and refer to them as rare haplotypes when they have an overall frequency less than 1%. Haplotypes having high support of being IBD between pairs of individuals are processed using DASH algorithm^6,7^ to identify overlapping regions between multiple individuals and thereby form clusters of individuals sharing the same genomic region IBD. These clusters of shared haplotypes were then tested for enrichment in patients using a permutation test. We applied this approach and found shared haplotypes between patients that have a distinct ethnic origin. This finding was unexpected given that their source ethnic groups were historically separated. Besides, linguistic and religious differences were likely hindrance to gene flow. We resolved this contradictory finding by reconstructing parameters of recent population history for these populations using haplotype sharing data. We found that source populations for patients exchanged a moderate level of recent gene flow in one direction and that IBD haplotypes carried by patients have local ancestry supporting this gene flow scenario. In sum, our study findings show that knowledge about the genetic ancestry of genomic loci and fine-scale details of recent population history is crucial to resolving contradictory findings regarding rare variant distribution among patients from multiethnic cohorts. This kind of knowledge is especially valuable when samples are coming from populations with mostly unknown and often complex population history.

## Results

### IBD mapping

In order to characterise rare variants shared between individuals in our study cohort, we analysed IBD haplotypes between samples. By looking for overlapping regions between pairwise IBD tracts (longer than 0.5 cM), we identified 103611 genomic regions shared by more than four individuals - clusters of individuals, or haplotype clusters. Our max(T) permutation test showed that the distribution of haplotype clusters between cases and controls in most cases could be explained by chance sampling; we found only two haplotypes, both rare, c2592 (0.8%) and c863 (0.7%), that showed statistically significant enrichment in patients (multiple testing corrected p-value less than 0.05, Table 2). Interestingly, these patients had a different ethnic origin (Table 2), and one would expect such distribution across ethnic groups only if haplotypes were widespread in the region.

**Table 2:** IBD clusters significantly enriched in patients with asthma.

Chromosome : Cluster	7 : c2592	15 : c863
Cluster size, number of haplotypes (overall frequency, %)	11 (0.8)	10 (0.7)
The genomic coordinates of the haplotype	52971553 : 54669568	51683053 : 53260405
Genetic length of haplotype, cM	1.233976	0.961478
Empirical p-value	0.000999	0.001499
Corrected empirical p-value	0.01299	0.04496
Bashkirs (haplotype IDs)	2 (17i.2, 9i.1)	1 (28i.1)
Russians (haplotype IDs)	6 (25i.1, 3AR.2, 4A.2, 50A.2, 78AR.1, 99A.1)	7 (137N.2, 19N.2, 206AR.2, 20AR.2, 26i.2, 39N.2, 79N.2)
Tatars (haplotype IDs)	3 (102N.2, 119A.2, 76AR.2)	2 (141N.2, 77A.2)

### Chromosome painting analysis and evidence for recent admixture

**Figure 2:**
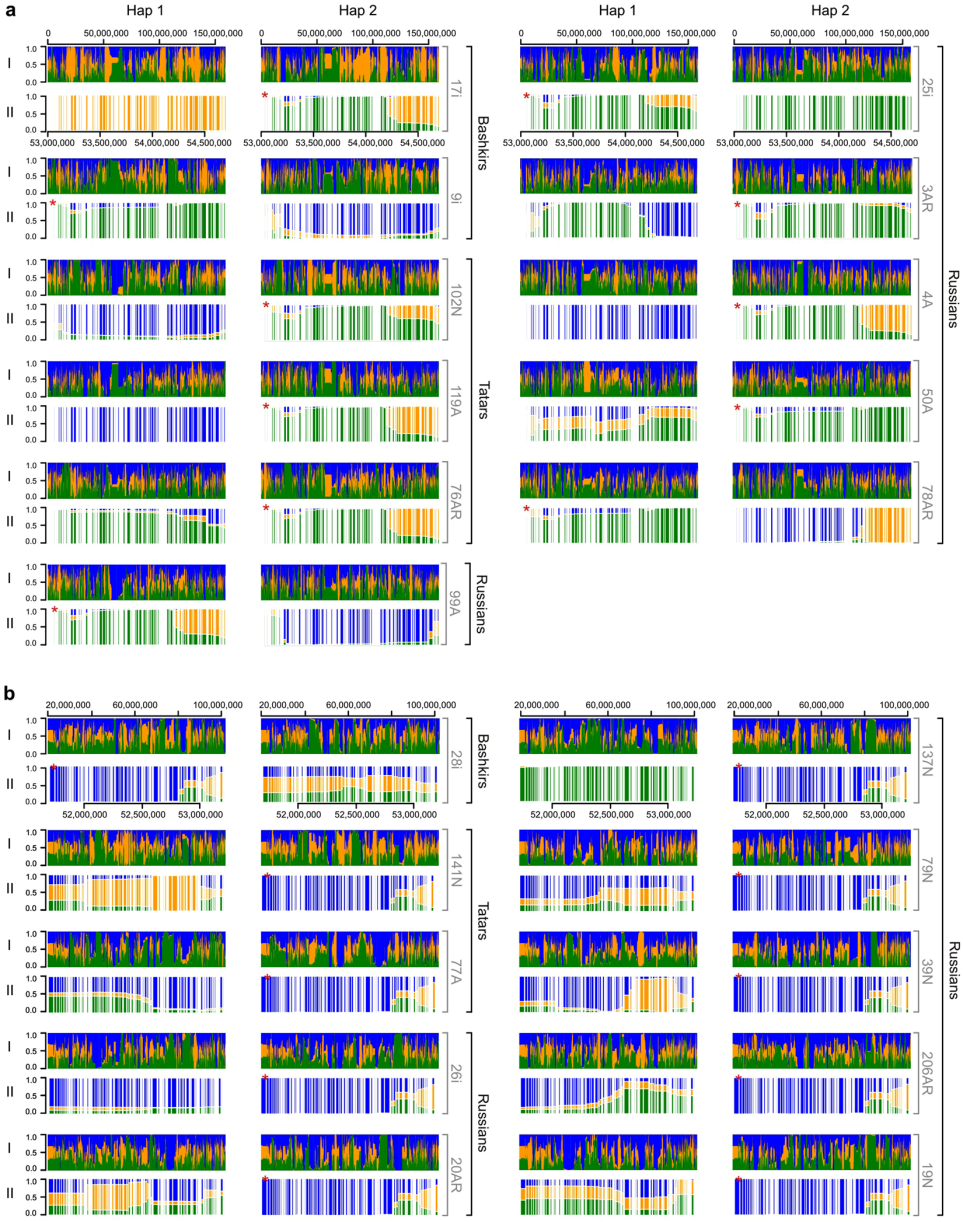
Chromosome chunk copying probability for (**a)** chromosome 7 in patients and the c2592 cluster region and for (**b**) chromosome 15 in patients and the c863 cluster region. (I) Barplot for the entire chromosome; (II) Barplot for the cluster region. Vertical bars filled in green, yellow and blue correspond to the probability of copying from Tatar, Bashkir, and Russian donors. For each patient both carrier (marked with a red asterisk) and non-carrier haplotypes are shown. Individual and population identifiers are shown at the right-hand side of each barplot. Hap1 and Hap2 indicate first and second homologous chromosomes of each individual.

To explain how patients from different ethnic groups (Table 2) came to share the same rare haplotypes (c2592 and c863), we analysed their phased haplotypes using chromosome painting/copying approach^15^. The idea was to test if the IBD haplotypes can be constructed from shorter chunks from a particular population. Our “chromosome painting” analysis showed that patients that carry the c2592 cluster (IBD tract) highly copy chromosomal chunks from Tatar donors (Fig. 2a) within the IBD haplotype and that carriers of the c863 cluster copy chromosomal chunks from Russian donors (Fig. 2b). These results suggest that IBD tracts in patients with different ethnic origin have (a) recent ancestor related to modern Tatars in case of the c2592 cluster, and (b) another recent ancestor related to modern Russians in case of the c863 cluster. This kind of IBD sharing is expected under inter-ethnic marriages, and we, therefore, studied recent population history parameters for the studied groups.

### Reconstructing population history

According to our ChromoPainter analysis, a recent gene flow between source populations (Russians, Bashkirs and Tatars) is a likely explanation for the shared risk haplotypes (c2592 and c863) between patients. To explore whether the three source populations exchanged recent gene flow, we reconstructed gene flow parameters for them. Since knowledge about past demographic history is critical to achieving accurate estimates of gene flow parameters, we first inferred ancestral dynamics of effective population size in the studied populations.

**Figure 3:**
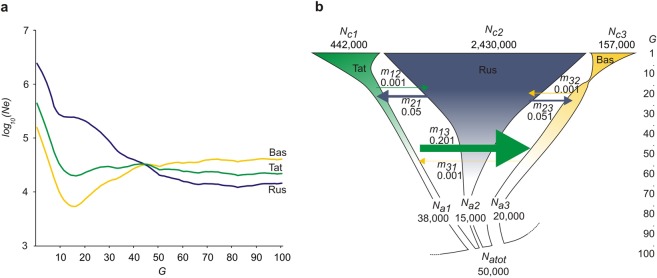
Population history parameters for the three source populations. (**a**) Effective population size history inferred using IBDNe method^16^. Populations analysed: Tat – Tatars, Rus – Russians, and Bas – Bashkirs. (**b**) Scheme summarising the inferred gene flow rates between populations and effective population size changes. Migration rates between studied populations inferred using *DoRIS* software^17^. Gene flow rates were inferred for each pair of populations using the demographic scenario ‘SplitExpConstAsymMig’. This scenario assumes an ancestral population of size *N*_*atot*_. The ancestral population splits *G* generations in the past and give rise to two populations. The two derived populations undergo a change in effective population size from *N*_*a1*_ and *N*_*a2*_ individuals to *N*_*c1*_ and *N*_*c2*_ individuals during the *G* generations since the split time. During this period, these populations exchange asymmetric gene flow at rates m_12_ and m_21_.

We applied IBDNe method^16^ on the observed IBD sharing data for each population to infer ancestral dynamics of effective population size (Fig. 3a). Here, we briefly outline features that deviate from the standard assumption of constant population size that is often used when inferring population genetic parameters. All the three populations experienced a period of rapid growth of effective population size, which was longer in duration and started earlier for Russians, at around 50 generations ago, compared to 10–15 generations for Bashkirs and Tatars. While Russians have two-epoch demographic history consisting of a constant population size phase and growth phase, we found that Tatars and Bashkirs experienced an additional phase of effective population size decline between 15 and 45 generations in the past. This signal of decline was more pronounced in Bashkirs than in Tatars. We next used these features of effective population size history as fixed parameters when applying the DoRIS approach to achieve more accurate estimates of gene flow parameters^17^ (Fig. 3b). Figure 3b shows the schematic summary of the inferred gene flow rates based on DoRIS along with effective population size changes inferred using the *IBDNe* method. DoRIS results suggest recent gene flow between populations that are asymmetric in intensity. Thus, we inferred a recent gene flow from Russians to both Tatars (m_21_~0.05) and Bashkirs (m_23_~0.051), but only trace level gene flow backwards (m_12_ = m_32_~0.001). This asymmetry in the directionality of gene flow generally agrees with the historical evidence that Russians migrated into Volga-Ural region from Russian principalities^18–20^. Next, we detected a considerable amount of gene flow (~0.201) from Tatars to Bashkirs, but only a weak signal in the backward direction. This strong signal of unidirectional gene flow agrees with the history of Tatar’s immigration into what is now modern Bashkortostan, the study region. In sum, we reconstructed fine-scale details of recent gene flow between study populations, which support our findings regarding the two rare haplotypes shared between ethnically diverse patients. Given this complex gene flow history, we next examined details of IBD haplotype sharing within and between source populations.

We classified DASH inferred clusters of shared haplotypes by size and ethnic composition based on whether haplotypes in a given cluster were carried by individuals of the same or different ethnic origin (Table 3). Haplotypes shared by four individuals (cluster size 4) were the most abundant class since for any given haplotype, we likely find only a few other matches in a given sample. At the same time, we likely find more matches for shorter and on average older haplotypes that tend to be more widespread geographically and shared by many populations (Table 3). This is why within each cluster size bin, we observe that haplotypes falling into multi-ethnic category are more abundant, and such haplotypes tend to be on average shorter in length (Table 4). Consistent with this expectation, we find that haplotypes that have more matches, i.e. have larger cluster size (9, 10, 11, for example), tend to have a shorter length (Table 4). Indeed, our putative risk haplotypes that are shared by ten and eleven patients (c863 and c2592, respectively) are relatively short in length (0.9 cM and 1.2 cM, respectively). They fit into the class of older and more widespread haplotypes that are observed more often among different ethnic groups. Thus, if a haplotype has properties of relatively old and widespread variant, then sampling it in at least one of the the three ethnic groups would be relatively high, unless it is linked to some deleterious mutation and present exclusively in affected individuals.

**Table 3:** IBD cluster counts binned by size (number of individuals) and ethnic composition.

Populations*	Cluster size															Total
	4	5	6	7	8	9	10	11	12	13	14	15	16	17	20	
Bas	2958	870	227	91	38	13	7	**3**	0	0	1	0	0	0	0	4208
Rus	3697	829	224	66	18	12	3	0	0	0	0	0	0	0	0	4849
Tat	1593	282	54	14	2	1	0	0	0	0	0	0	0	0	0	1946
Bas_Rus	6627	2002	767	357	128	58	17	5	1	0	0	0	0	0	0	9962
Bas_Tat	11207	4096	1646	853	396	212	104	33	25	14	4	0	1	0	0	18591
Rus_Tat	15982	5757	2344	1190	472	273	108	29	11	6	2	1	0	0	0	26175
Bas_Rus_Tat	15367	8784	5327	3640	2042	1342	745	346	158	74	39	11	2	1	1	37879
Total	57431	22620	10589	6211	3096	1911	984	416	195	94	46	12	3	1	1	103610

*Bas, Rus, and Tat indicate Bashkir, Russian and Tatar ethnic origin for individuals carrying haplotypes.

**Table 4:** Average length (cM) of haplotypes in each cluster size bin.

Populations*	Cluster size															Total
	4	5	6	7	8	9	10	11	12	13	14	15	16	17	20	
Bas	3.8	3.3	3.0	3.2	2.8	3.1										3.6
Rus	2.5	2.4	2.3	2.3	2.2	2.0										2.5
Tat	3.1	2.7	2.7	2.3												3.0
Bas_Rus	2.6	2.5	2.4	2.3	2.3	2.1	2.0									2.5
Bas_Tat	3.1	2.9	2.7	2.6	2.6	2.5	2.6	2.3	2.4	2.3						3.0
Rus_Tat	2.6	2.4	2.3	2.2	2.2	2.1	2.0	2.1	2.0							2.5
Bas_Rus_Tat	2.6	2.5	2.4	2.3	2.3	2.1	2.2	2.1	2.0	2.1	2.0					2.5
Total	2.9	2.7	2.5	2.5	2.4	2.5	2.3	2.4	2.1	2.1						2.8

Average values were calculated only for clusters with the number of haplotypes >10. *Bas, Rus, and Tat indicate Bashkir, Russian and Tatar ethnic origin for individuals carrying haplotypes.

Finally, we interrogated the GRASP^21^ database for known asthma associated genetic variants that overlap with our putative risk haplotypes c2592 and c863 (Table 5). Genomic region for c2592 haplotype contained variants, such as rs6944870 (p = 2.6E-04) and rs17560456 (p = 3.9E-04)^22^, that are associated with decreased FEV1/FVC ratio. This airway obstruction phenotype is characteristic for asthma and chronic obstructive pulmonary (lung) disease (COPD). The genomic region for c863 haplotype contained variants, such as rs17525472 (p = 1.50E-06, located in gene SCG3)^23^, rs1063902 (p = 3.6E-04), rs4774612 (p = 6.6E-03) (MYO5C), rs2445743 (p = 7.2E-03) (GLDN), and rs4238384 (p = 8.7E-03) (GNB5)^11^ reported in connection with asthma association, but not reaching currently adopted genome-wide significance threshold of 8.7E-07. Since the interrogated GRASP database contained a large number of SNPs for asthma (41617 SNPs) and the SNPs were clustered in patches, we estimated whether the overlaps with our IBD haplotypes could be explained by chance (Details in Materials and Methods). Briefly, we generated 100000 random haplotype pairs matching in size our IBD tracts and counted number of times we observed 2 and 7 or more SNPs from the GRASP database. We estimate that the probability of this event is ~0.005. This estimate suggests that our observed matches with GRASP SNPs are unlikely to be by chance.

**Table 5:** Previously identified asthma-associated loci within detected haplotypes.

Chromosome: Cluster	SNP, start:end	Position, start:end	SNP included associated with asthma*	
			p < 1·10^−2^	p < 1·10^−7^
7 : c2592	rs11773742: rs12531167	52971553: 54669568	rs6944870, rs17560456	NA
15 : c863	rs2445743: rs4776110	51683053: 53260405	rs17525472, rs1063902, rs4774612, rs2445743, rs4238384	NA

*GRASP Build 2.0.0.0 data^21^.

## Discussion

We report evidence for two rare IBD haplotypes that are strongly enriched in asthma patients and that descend from ancestors with different ethnic origin. Importantly, we did not observe these haplotypes in the source populations: Tatars, Russians and Bashkirs. In the absence of evidence for historical gene flow between ethnic groups, this finding would be difficult to interpret and could be interpreted as potential mislabeling. Although pairwise IBD haplotypes can be observed between populations across Europe^24^ and beyond^10^, more often such rare haplotypes are confined to one population, and the rate of between population sharing depends on details of recent shared demographic past.

In order to interpret our findings regarding these IBD haplotypes in patients with different ethnic origin, we first applied chromosome copying analysis and found that patients derived their IBD haplotypes through admixture from one source population. This finding implied a recent gene flow between studied populations, and we next carried out the reconstruction of recent population history. To achieve accurate inference, we first inferred dynamics of effective population size for the studied populations, which is critical to reconstructing gene flow parameters^17^. By accounting for effective population size dynamics, we estimated gene flow rates between populations. Inferred parameters suggested a recent history of asymmetric gene flow between populations. This detailed insight into population history helped us to explain why we observe the two rare haplotypes among patients of different ethnic origin and ensure that it is not an artifact due to a mislabeled ethnic origin.

By ensuring that our findings are (a) extreme, i.e. have significant p-values, and (b) plausible in light of the observed population level patterns, we next tested whether our reported genomic segments fall within previously reported genomic loci associated with asthma.To date, a number of genome-wide association studies reported genomic loci associated with asthma: based on patients of European^11,23,25–42^, African^43,44^, Asian^45,46^, Latino^47–50^ ancestry, as well as multiethnic cohorts^49,51–55^ or integrated data from multiple GWAS studies^21^. We found that our reported segments overlap with earlier published asthma-associated loci discovered on samples having European ancestry (Table 5). Taken together our findings suggest that the identified haplotypes might carry putative rare variants shared with European populations. This overlap with European populations is consistent with the published estimates of ancestry proportions for Russians, Tatars and Bashkirs based on ADMIXTURE analysis^10^. According to ADMIXTURE results, 75 per cent of ancestry in Russians, 54 per cent in Tatars and 37 per cent in Bashkirs have “dark blue” ancestry represented primarily in European populations^10^.

In conclusion, in the absence of resequencing data for underrepresented populations, available chip-genotype data and haplotype-based methods provide valuable insight into the distribution of rare variants. Because population history for underrepresented populations is generally poorly understood, inference about population history is imperative and should accompany gene mapping studies^4^. This is especially true when a study population has a complex population history with admixture. Since there is still bias towards patients of European descent^56–59^, more genomic data is needed from underrepresented populations to assess missing diversity of ancestry-specific variants and potential implications for disease risk estimates and transferability issue^56–59^.

## Methods

### Samples and quality control

We retrieved Illumina 650 k Bead Chip genotypes on 342 asthma patients and 359 healthy donors generated by the GABRIEL Consortium^11^. Altogether 582,892 markers were available upon data retrieval from the European Genome-phenome Archive (https://www.ebi.ac.uk/ega/home). Individuals with more than 1.5% missing genotypes were removed from the dataset. Only markers with a 97% genotyping rate and minor allele frequency (MAF) > 1% were retained. The absence of cryptic relatedness corresponding to first- and second-degree relatives in our dataset was confirmed using the KING software^60^.

After filtering the genotypes for quality, we explored genetic distances between individuals using MDS plot and removed outliers (see Supplementary Fig. S1).

The filtering steps resulted in a dataset of 673 individuals (330 cases and 343 controls) and 525,296 SNPs available for all the downstream analyses. Genetic distances between SNPs in centimorgans were incorporated from the GrCh37 genetic map generated by the HapMap project^61^. Genotypes were phased using BEAGLE version 4.0^14^.

### Detecting pairwise IBD segments

We used the refined IBD algorithm^14^ implemented in the BEAGLE 4.0 software to detect extended chromosomal tracts (>0.5 cM) that are IBD between pairs of individuals. We ran BEAGLE 4.0 with the following parameters: ibdtrim = 20 window = 700 overlap = 300 nthreads = 10 niterations = 10 ibdlod = 4.5 ibdcm = 0.5. Altogether, we detected 1.4 millions of pairwise IBD segments that were used for clustering and downstream association testing. To perform demographic inference, we first generated IBD sharing dataset using IBDSeq software^62^ and then used this precomputed dataset for both DoRIS and IBDNe analyses. IBDSeq was run using the following flags: nthreads = 40 minibd = 2.

### Detecting IBD clusters

We examined whether IBD tracts shared by a pair of individuals were also shared by other samples in the dataset using DASH algorithm^7^. This algorithm clusters individuals based on overlapping IBD segment. These segments on average represent the recent haplotype sharing and could thus serve as proxies for recent variants that are generally rare (<1%) and difficult to detect otherwise. Most of the clusters (103593 out of 103610) detected in our sample were observed in less than 14 individuals (Table 3) and hence could be classified as rare 14/673 * 2 ~ 0.01. We detected 103593 IBD clusters altogether and tested them for association with asthma phenotype.

### Assessing significance of enrichment

After identifying the clusters of individuals that carry overlapping segments, we searched for clusters that are enriched in asthma patients. We used the max(T) permutation test in the PLINK package^63^ to assess enrichment. This permutation procedure allows controlling the family-wise error in multiple testing settings by calculating corrected p-values. The corrected p-value reflects the chance of seeing a test statistic as large as we observe (the observed distribution of haplotypes in the tested cluster), given we have performed as many permutation tests as 103611 clusters (i.e. independent permutation runs for all the 103611 clusters). Out of 103611 observed clusters, only two showed multiple-testing corrected empirical p-value less than 0.05 (Table 2). We then interrogated GRASP database to test whether genomic regions for our clusters overlap with the previously published genomic loci associated with asthma^21^. We queried the GRASP database using two p-value thresholds: (a) 1 × 10^−7^ (Table 5), which is recommended for genome-wide association studies^64^ and 0.01, which is a more relaxed threshold to report putative associations with weak statistical evidence. Our GRASP query identified 2 and 7 SNPs overlapping with our rare haplotypes c2592 and c863, respectively (Table 5). Because of the large number of the asthma-associated SNPs in the GRASP database (41617), we calculated the probability of encountering our observation by chance using our custom python RandHaps tool (see Electronic Supplementary Material). We used the map files for our Illumina dataset to generate random haplotype pairs within the boundaries of 22 chromosomes. Because our observed IBD tracts (c2592 and c863) had the nearly similar physical size of 1698015 b.p. and 1577352 b.p, we took average size to generate the random haplotype. Thus, the probability of our observed event of having at least 2 SNP (as in c2592) and 7 SNPs (as in c863) was ~0.005 over many repetitions of this random experiment.

### Chromosome painting analysis

We used the ChromoPainter algorithm^15^ based on Li and Stephen’s^65^ copying model, to estimate the ancestry of each chromosomal chunk. The ChromoPainter uses a Hidden Markov Model to reconstruct a sampled haplotype as it would be constructed by a copying process from all other haplotypes in the donor populations. In our study, the likely ancestry of each chromosomal chunk was obtained by estimating copying probabilities from 3 donor populations (Bashkirs, Tatars and Russians). Thus, to “paint” chromosomes, we prepared three donor samples that included 60 individuals from each population.

### Estimation of the effective population size and migration rate

We used DoRIS software^17^ to infer gene flow parameters between studied populations. DoRIS can be used to jointly infer most likely gene flow and demographic history parameters based on IBD sharing data. We precomputed the effective population size (Fig. 3a) using IBDNe software^16^ that uses information on IBD sharing. We then fixed the precomputed parameters of effective population size history and run DoRIS to infer only gene flow rates. We run DoRIS with the following flags: “–DemographicModel SplitExpConstAsymMig”. The selected demographic model assumes an ancestral population of constant size *N*_*atot*_. This ancestral population splits *G* generations in the past and results in two populations whose sizes independently fluctuate from *N*_*a1*_ and *N*_*a2*_ individuals to *N*_*c1*_ and *N*_*c2*_ individuals during *G* generations. During this period, the two populations exchange gene flow at rates m_12_ and m_21_. This demographic model assumes only two populations. We, therefore, inferred gene flow parameters for our three populations using different pairwise combinations: Bashkirs-Russians, Bashkirs-Tatars, Tatars-Russians. The command to run DoRIS was: “–DemographicModel: SplitExpConstAsymMig; pop1current: fixed, pop1ancestral: unfixed, pop2current: fixed, pop2ancestral: unfixed, ancestraltot: unfixed, generation: unfixed, m12: unfixed, m21: unfixed”.

### *IBDMig* python tool to classify shared haplotypes using population labels

We implemented a Python tool IBDMig (see Electronic Supplementary Material) to sort IBD clusters by genetic length, cluster size (number of individuals that share the haplotype) and population source of the individual carrying the haplotype in the cluster. This tool was used to generate Tables 3 and 4 in this study. To generate Table 3, IBDMig sorts clusters by size and population source of individuals that carry the haplotype in the given cluster. Additionally, IBDMig can be used to estimate the average length of haplotypes in each cluster size category as shown in Table 4. In combination, data in Tables 3 and 4 are useful to explore within population and between population haplotype sharing that reflects recent demographic and migration history. We run the IBDMig tool with the following parameters:./ibdmig.py 22 ibdmig.list, where 22 is the number of chromosomes; ibdmig.list is the file containing a list of individuals with individual ID, population, and phenotype.

## Supplementary information

Reconstructing recent population history while mapping rare variants using haplotypes

## Data Availability

The Illumina 650 k genotyped dataset analysed in this study was previously published in^11^ and is publicly available through European Genome-phenome Archive (https://www.ebi.ac.uk/ega/home).
