# Supplementary material for: Reconstructing recent population history while mapping rare variants using haplotypes
Source: Sci Rep. 2019 Apr 10;9:5849. doi: 10.1038/s41598-019-42385-6 (PMC6458133; doi:10.1038/s41598-019-42385-6)
Supplement: Supplementary file 1 — Reconstructing recent population history while mapping rare variants using haplotypes [file 41598_2019_42385_MOESM1_ESM.pdf]

SUPPLEMENTARY INFORMATION

Reconstructing recent population history while mapping rare variants using haplotypes

Ural Yunusbayev[1,4,*,+], Albert Valeev[1], Milyausha Yunusbaeva[1,2], Hyung Wook Kwon[4], Reedik Mägi[3], Mait Metspalu[3], Bayazit Yunusbayev[3,+]

[1]Ufa Federal Research Center of the Russian Academy of Sciences, Institute of Biochemistry and Genetics, Ufa, 450054, Russia
[2]Bashkir State Pedagogical University n. a. M. Akmulla, Department of Genetics, Ufa, 450000, Russia
[3]University of Tartu, Institute of Genomics, Tartu, 51010, Estonia
[4]Incheon National University, Incheon, 22012, South Korea
[*]corresponding author uralub@gmail.com
[+]these authors contributed equally to this work

Table S1. Haplotype clusters associated with asthma

Cluster ID	Cluster size (number of individuals)	Chromosome	Segment's start / end positions (bp)	Segment's length (cM)	LOD of IBD segments	p-value	Corrected p-value
c2592	11	7	52971553:54669568	1.233976	4.5	0.000999	0.01299
c863	10	15	51683053:53260405	0.961478	4.5	0.001499	0.04496

Table S2. Haplotype cluster c2592

Haplotype ID	Ethnos	Status (1 - control, 2 - patient)	Age	Age of onset status	Total IgE (IU/ml)	IgE measure age	Asthma status
17i.2	Bas	2	9	1,5	310	8	child
9i.1	Bas	2	3	2	418	3	child
25i.1	Rus	2	15	12	128	15	child
3AR.2	Rus	2	5	3	480	5	child
4A.2	Rus	2	30	21	420	29	adult
50A.2	Rus	2	25	9	520	25	child
78AR.1	Rus	2	9	5	420	8	child
99A.1	Rus	2	50	17	430	49	child
102N.2	Tat	2	14	4	651	13	child
119A.2	Tat	2	59	47	250	58	adult
76AR.2	Tat	2	16	8	444	14	child

Table S3. Haplotype cluster c863

Haplotype ID	Ethnos	Status (1 - control, 2 - patient)	Age	Age of onset status	Total IgE (IU/ml)	IgE measure age	Asthma status
28i.1	Bas	2	15	7	240	14	child
137N.2	Rus	2	17	11	593	16	child
19N.2	Rus	2	11	8	380	8	child
206AR.2	Rus	2	13	9	560	12	child
20AR.2	Rus	2	16	4	500	15	child
26i.2	Rus	2	13	3,5	106	12	child
39N.2	Rus	2	11	7	139	11	child
79N.2	Rus	2	5	2	70	5	child
141N.2	Tat	2	5	1,5	33	5	child
77A.2	Tat	2	58	51	220	58	adult

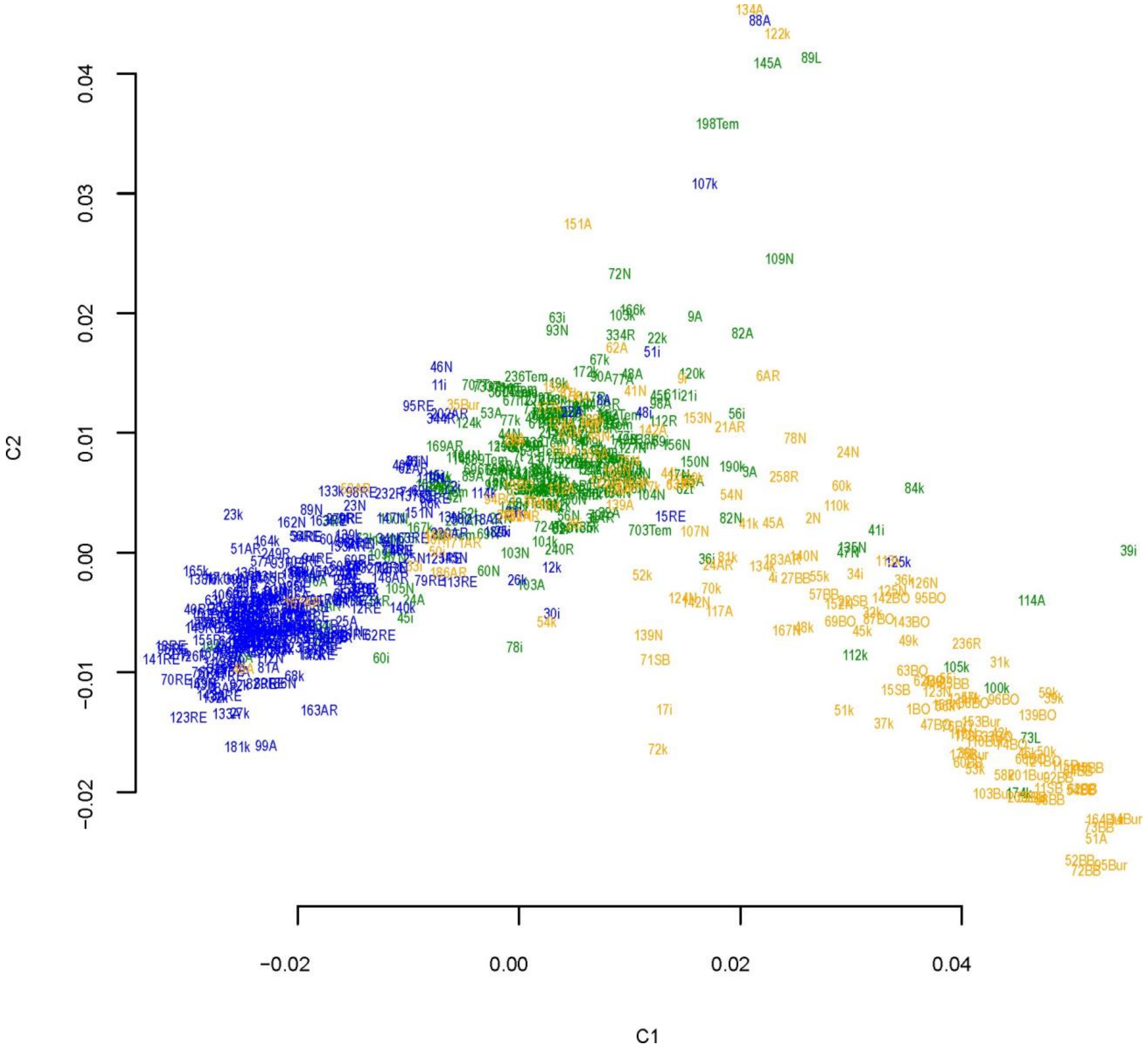

Figure S1. MDS plot of genome-wide IBS distances between individuals of the total dataset. C1 - position on the first dimension, C2 - position on the second dimension. Color indicates ethnic origin: orange - Bashkirs; blue - Russians, green - Tatars.
